# Epigenetic signature of Gleason score and prostate cancer recurrence after radical prostatectomy

**DOI:** 10.1186/s13148-016-0260-z

**Published:** 2016-09-15

**Authors:** Milan S. Geybels, Jonathan L. Wright, Marina Bibikova, Brandy Klotzle, Jian-Bing Fan, Shanshan Zhao, Ziding Feng, Elaine A. Ostrander, Daniel W. Lin, Peter S. Nelson, Janet L. Stanford

**Affiliations:** 1 Division of Public Health Sciences, Fred Hutchinson Cancer Research Center, 1100 Fairview Avenue North, Seattle, WA 98109-1024 USA; 2 Department of Epidemiology, GROW School for Oncology and Developmental biology, Maastricht University, Maastricht, The Netherlands; 3 Department of Urology, University of Washington School of Medicine, Seattle, WA USA; 4 Illumina, Inc., San Diego, CA USA; 5 Biostatistics & Computational Biology Branch, National Institute of Environmental Health Sciences, Research Triangle Park, Durham, NC USA; 6 Department of Biostatistics, MD Anderson Cancer Center, Houston, TX USA; 7 Cancer Genetics and Comparative Genomics Branch, National Human Genome Research Institute, NIH, Bethesda, MD USA; 8 Division of Human Biology, Fred Hutchinson Cancer Research Center, Seattle, WA USA; 9 Division of Clinical Research, Fred Hutchinson Cancer Research Center, Seattle, WA USA; 10 Department of Medicine, University of Washington School of Medicine, Seattle, WA USA; 11 Department of Epidemiology, University of Washington School of Public Health, Seattle, WA USA; 12 Current address: AnchorDx Corp., Guangzhou, 510300 People’s Republic of China

**Keywords:** Clinically localized prostate cancer, Tumor tissue, DNA methylation, Gene expression, Risk prediction for prognosis, Genome-wide profiling, Elastic net regularization

## Abstract

**Background:**

Identifying the subset of patients with clinically localized prostate cancer (PCa) at the highest risk of recurrence remains challenging, and better prognostic markers are needed. Gleason score is the best predictor of PCa aggressiveness and prognosis. In the present study, we generated an epigenetic signature based on high versus low Gleason score tumors and evaluated its ability to predict recurrence after radical prostatectomy.

**Methods:**

Genome-wide DNA methylation data from The Cancer Genome Atlas (TCGA; no. of patients = 333) and the elastic net method were used to generate an epigenetic signature by contrasting patients with high (8–10) versus low (≤6) Gleason score tumors. The signature was then tested in a cohort of 523 patients with clinically localized disease who had radical prostatectomy. Samples taken from the primary tumor were used for DNA methylation and mRNA expression profiling. Patients were followed for PCa recurrence on average for 8 years after diagnosis.

**Results:**

The epigenetic signature includes 52 differentially methylated CpG sites. In the testing cohort, the signature was associated with poorer recurrence-free survival (hazard ratio per 25 % increase = 1.78; 95 % confidence interval 1.48, 2.16). The signature significantly improved the area under the curve (AUC) for PCa recurrence compared to clinical-pathological parameters alone, particularly among patients diagnosed with Gleason score 7 tumors (0.64 vs. 0.76, *P* = 1.34E−4). Results were comparable for patients with Gleason 3 + 4 and those with 4 + 3 tumors. Gene Set Enrichment Analysis showed that higher levels of the signature were associated with increased expression of genes related to cell cycle proliferation and decreased expression of androgen-responsive genes.

**Conclusions:**

This report shows evidence that DNA methylation patterns measured in prostate tumor cells are predictive of PCa aggressiveness. The epigenetic signature may have clinical utility to improve prognostication particularly in patients with intermediate Gleason score 7 tumors.

**Electronic supplementary material:**

The online version of this article (doi:10.1186/s13148-016-0260-z) contains supplementary material, which is available to authorized users.

## Background

Prostate cancer (PCa) is the most common solid tumor in men [1]. While many PCa patients are diagnosed with indolent disease that is unlikely to progress even if left untreated, other patients will have aggressive tumors that may become life-threatening [2–4]. Although current clinical and pathological measures such as Gleason score (tumor grade), disease stage, and prostate-specific antigen (PSA) level provide important prognostic information, they do not accurately predict an individual patient's risk of progression, and better markers to aid prognostication are needed [4, 5].

DNA methylation is an epigenetic alteration that occurs at CG dinucleotides (CpG sites) [6]. The human DNA methylome is generated in a programmed manner during normal development and methylation patterns change as a result of aging [7]. Patterns of DNA methylation record a remarkable breadth of information about cells, including their chronological age, developmental history, and differentiation potential [8, 9]. In PCa, there is substantial heterogeneity in tumor DNA methylation profiles [10], and these epigenetic changes may also predict PCa aggressiveness. Some previous studies showed that differentially methylated CpG sites in specific genes are associated with more aggressive and advanced PCa, but most of these investigations focused on selected candidate genes and findings require validation [11–18].

In this study, we generated an epigenetic (DNA methylation) signature for use as a prognostic classifier in PCa. Because Gleason score is the best predictor of PCa prognosis [19], we generated the signature by contrasting patients with high (8–10) versus low (≤6) Gleason score tumors. The signature was then tested for its ability to predict recurrence in a validation cohort of patients with clinically localized PCa who had radical prostatectomy.

## Methods

### Study population

The Fred Hutchinson (FH) Cancer Research Center cohort includes 565 PCa patients who underwent radical prostatectomy as primary therapy for clinically localized adenocarcinoma of the prostate. These patients were previously enrolled in population-based studies of PCa (diagnosed in 1993–1996 or 2002–2005) [20, 21]. Clinical information and vital status were collected from the Seattle-Puget Sound Surveillance, Epidemiology, and End Results (SEER) Program cancer registry. Prostate cancer recurrence status was determined from two detailed follow-up surveys that were completed by patients in 2004–2005 and in 2010–2011, with review of medical records or physician follow-up as needed. A patient was considered to have disease recurrence based on (1) a post-surgery PSA value of 0.2 ng/mL or greater; (2) metastatic progression on a bone scan, MRI, CT, or biopsy; and (3) PCa-specific death. The mean follow-up time for recurrence was 8 years. The Institutional Review Board of the Fred Hutchinson Cancer Research Center approved the study, and all participants signed informed consent statements.

### DNA and RNA isolation

Formalin-fixed paraffin-embedded (FFPE) prostate tumor tissue blocks were obtained from radical prostatectomy specimens and used to make hematoxylin and eosin-stained slides, which were reviewed by a PCa pathologist to confirm the presence and location of prostate adenocarcinoma. For each patient, two 1-mm tumor tissue cores from the dominant lesion that were enriched with ≥75 % tumor cells were taken for DNA and RNA purification. The RecoverAll Total Nucleic Acid Isolation Kit (Ambion, Applied Biosciences, Austin, TX) was used to extract DNA. The RNeasy® FFPE Kit (Qiagen Inc., Valencia, CA) was used to isolate RNA. DNA and RNA samples were shipped to Illumina (Illumina, Inc., San Diego, CA) for DNA methylation and mRNA expression profiling.

### Molecular profiling and data preprocessing

Tumor DNA was bisulfite converted. The Infinium® HumanMethylation450 BeadChip array (Illumina) was used for methylation profiling. Methylation data were normalized using subset-quantile within array normalization (*minfi* in Bioconductor) [22], and batch effects were removed using ComBat [23]. Methylation *β* values were calculated, which represent the percentage of DNA methylation at a CpG site. Genome annotation was based on the Illumina Manifest, and a gene promoter region was defined as TSS1500, TSS200, 5′UTR, or 1stExon. Across the 96-well plates, we incorporated blind duplicate (*n* = 16) and replicate (*n* = 2) samples. A sample was excluded if less than 95 % of the CpG sites for that sample on the array were detected with a detection *P* value (probability of a CpG being detected above the background level defined by negative control probes) of <0.05, and 42 samples were excluded. Further, CpG sites with a detection *P* value of >0.01 were excluded. Correlation coefficients for duplicate samples were 0.96–0.99. The correlation coefficient for the replicate samples was 0.99. There were 523 patients with DNA methylation data.

The HumanHT-12 v4.0 Whole-Genome DASL HT Assay (Illumina) was used for gene expression profiling. Low-quality probes were filtered out (illuminaHumanWGDASLv4.db package in Bioconductor). Gene expression data were quantile normalized and log_2_ transformed. Batch effects were removed using ComBat [23]. Genome annotation was based on the Illumina protocol. Blind duplicate samples from 11 patients had correlations ranging from 0.98 to 0.99, and replicate samples from two patients had mean correlations of 0.99. There were 469 patients with mRNA expression data, and these patients also had DNA methylation data.

### The Cancer Genome Atlas DNA methylation data

Level 1 Infinium HumanMethylation450 data were downloaded from The Cancer Genome Atlas (TCGA) data portal (https://tcga-data.nci.nih.gov/tcga/). The TCGA dataset includes 333 PCa patients who received radical prostatectomy [10]. The number of patients with Gleason ≤6, 7(3 + 4), 7(4 + 3), and 8–10 tumors is 65, 102, 78, and 88, respectively. Data on long-term patient outcomes are not available in TCGA [10]. Gene expression data from TCGA were downloaded from the Cancer Browser (https://genome-cancer.ucsc.edu/).

### Statistical data analysis

Genome-wide DNA methylation data from TCGA were used to generate an epigenetic signature of Gleason score by contrasting patients with high (8–10) versus low (≤6) Gleason score tumors. The signature was built using the elastic net method (*glmnet* in R), which is a regularization and variable selection method for high-dimensional data [24]. This approach is a combination of traditional LASSO and ridge regression methods, emphasizing model sparsity while appropriately balancing the contributions of correlated variables [25]. All CpG sites were used as input for the analysis. Fivefold cross-validation and the area under the curve (AUC) criterion were used to determine the optimal tuning parameter *λ* for classification. After variable selection using elastic net, the signature was calculated as follows: signature_*i*_ = ∑_*g* = 1_^*n*^*β*_*g*_×*X*_*gi*_, where *g* is the marker (i.e., CpG site); *n* is the number of markers; *β*_*g*_ is the elastic net coefficient for marker *g*; and *X*_*gi*_ is the methylation value for marker *g* and patient *i*.

The epigenetic signature was next evaluated for its ability to predict PCa recurrence in the FH cohort. Kaplan-Maier analysis and Cox regression models were used to examine the association between quartiles of the signature and recurrence-free survival. Hazard ratios (HRs) and 95 % confidence intervals (CIs) were calculated. A receiver operating characteristic (ROC) analysis was performed to evaluate the ability of the signature to distinguish patients with no evidence of recurrence from those who developed recurrence. A likelihood ratio test was used to compare a model that included the standard clinical-pathological parameters Gleason score (≤6, 7(3 + 4), 7(4 + 3), and 8–10), pathological stage (local: pT2, N0/NX, M0; regional: pT3-T4 and/or N1, M0), and diagnostic PSA level (1-unit increase), with a model that included these clinical-pathological variables and the epigenetic signature.

Tumor mRNA expression data were then used to study correlations between methylation levels of individual CpG sites in the signature and expression levels of corresponding genes. Pearson correlation coefficients were used. Next, Gene Set Enrichment Analysis (GSEA) was performed [26]. First, correlations between the signature and genome-wide gene expression levels were evaluated. The genes were then pre-ranked according to Pearson correlation and false discovery rate (FDR) *Q* value. Genes with a *Q* value of <0.05 were used as input for GSEA. For those genes with multiple transcripts, the average mean correlation across transcripts was calculated. GSEA was run with 1000 gene set permutations to calculate FDR *Q* values. We tested for the enrichment of “hallmark” gene sets, which have been shown to reduce variation and redundancy thereby providing more refined and concise inputs for GSEA [27]. The GSEA results were shown using normalized enrichment scores (NES), which is a value assigned to each gene set after normalization across all analyzed gene sets. NES is calculated by the following formula: NES = actual ES/mean (ESs against all permutations of the dataset) [26, 28]. All statistical analyses were done using R.

## Results

**Fig. 1:**
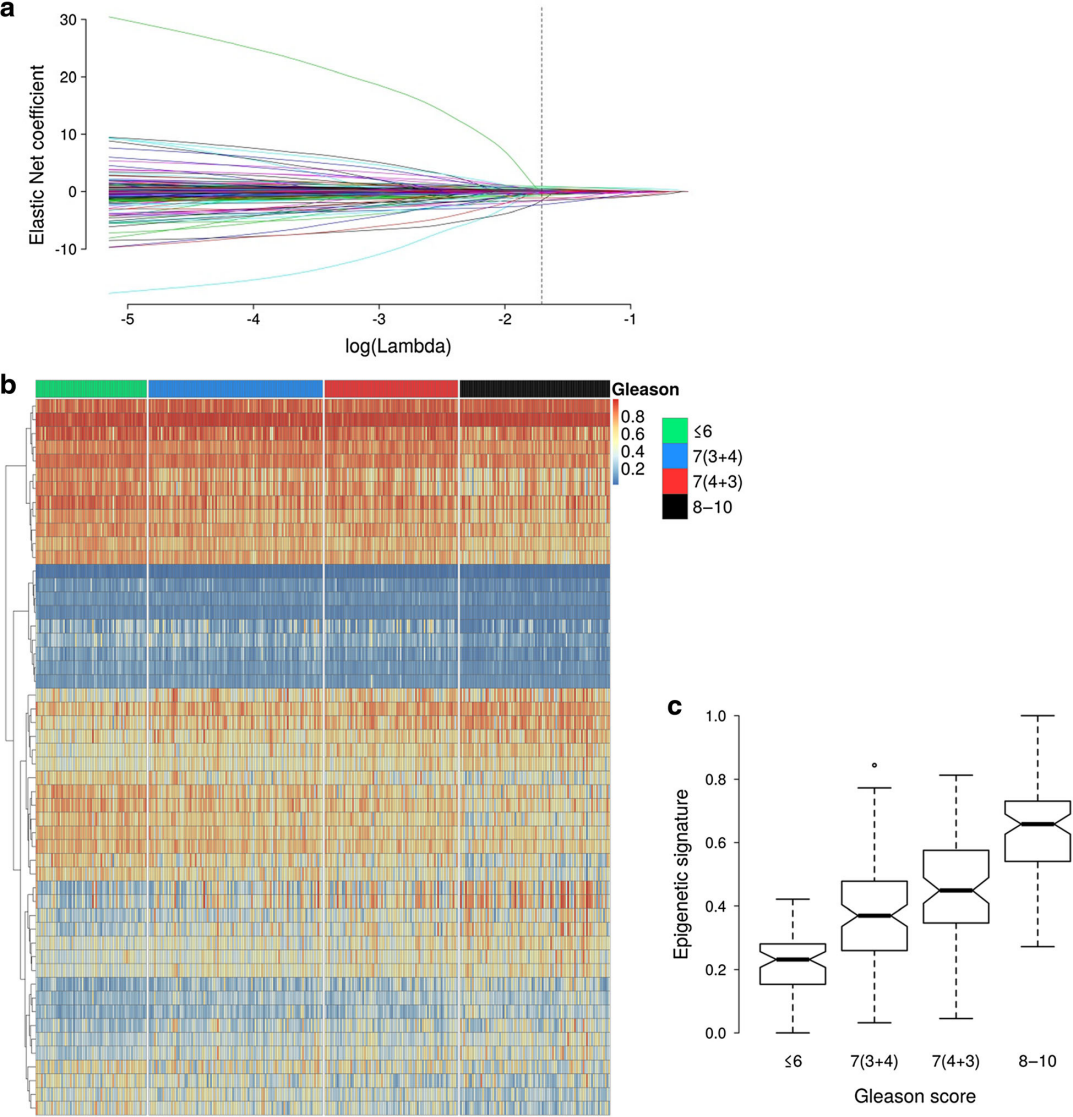
Epigenetic signature of Gleason score in The Cancer Genome Atlas. **a** Variable selection using elastic net. Each *curve* in the figure corresponds to a single CpG site. It shows the path of its coefficient (*y-axis*) against the log(lambda) or tuning parameter (*x-axis*). The *vertical dashed line* represents the optimal log(lambda) for classifying high (8−10) versus low (≤6) Gleason score tumors, which was identified using cross-validation. Based on an optimal log(lambda) of −1.7061, 52 CpG sites were selected. These 52 CpGs and their elastic net coefficients were then used to calculate the epigenetic signature as described in the “Methods” section. **b** Heatmap of the 52 CpG sites that were selected using elastic net. The *rows* of the heatmap are the CpG sites, and the *columns* are the tumor samples. The samples were grouped by Gleason score. Methylation *β* values (*range 0−1*) were used, and the highest methylation levels are shown in *red*. The number of patients with Gleason ≤6, 7(3 + 4), 7(4 + 3), and 8−10 tumors is 65, 102, 78, and 88, respectively. The *rows* were clustered based on Euclidean distance. **c** Box plots of the epigenetic signature (*y-axis*) for patients with different Gleason scores. The signature is presented as a proportion. Higher Gleason scores were associated with higher levels of the signature. The same patients as in Fig. 1b were used

The epigenetic signature of Gleason score generated in the TCGA cohort includes 52 differentially methylated CpG sites (Fig. 1a, b). Patients with Gleason score 7 tumors had intermediate levels of the signature compared to Gleason ≤6 and 8–10 tumors (Fig. 1c). Twenty-one CpGs in the signature had higher methylation levels in Gleason 8–10 tumors (Table 1). Thirty-six CpGs are in genes (32 unique genes), and 18 are in gene promoter regions. In the FH cohort (Table 2), higher levels of the signature correlated with increasing Gleason score (correlation  = 0.33; *P* = 2.11E−14) (Additional file 1: Figure S1).

**Table 1:** Fifty-two CpG sites included in the epigenetic signature

CpG ID	Chr.	Gene name	Genetic location	Epigenetic location	Mean β Gleason ≤6	Mean β Gleason 8−10	Mean β difference	Elastic Net coefficient
cg10145000	1				0.44	0.31	0.12	−0.0043
cg00043324	1				0.87	0.90	0.03	0.2022
cg00506866	2	*RRM2*	Body	Island	0.16	0.11	0.04	−0.2591
cg02601249	2			S_Shore	0.80	0.62	0.17	−0.1426
cg15454811	2				0.82	0.65	0.17	−0.1904
cg09741917	2	*VWA3B*	TSS1500	N_Shore	0.49	0.63	0.14	0.3933
cg13607230	2	*MFSD9*	Body	N_Shore	0.43	0.56	0.13	0.5376
cg17353895	2	*ANO7*	3'UTR;Body		0.65	0.76	0.10	0.5339
cg05287437	3	*GALNTL2*	TSS1500		0.77	0.63	0.14	−1.5620
cg13333267	3	*SEMA3F*	5'UTR	S_Shelf	0.48	0.59	0.11	0.0180
cg13320202	3	*ATXN7*	Body	S_Shore	0.48	0.36	0.12	−0.0575
cg10523671	3	*SLC15A2*	TSS1500		0.45	0.62	0.17	0.3300
cg02034887	3	*SLC15A2*	TSS200		0.27	0.40	0.14	0.0982
cg05962239	3	*MME*	5'UTR;1stExon	S_Shore	0.24	0.14	0.10	−0.2355
cg27473997	4	*USP17*	TSS200		0.71	0.55	0.17	−0.2529
cg13432241	4	*KIAA0922*	Body	S_Shore	0.61	0.44	0.17	−0.4088
cg08415137	5				0.13	0.09	0.04	−0.0529
cg01106114	5	*FOXI1*	TSS1500	N_Shore	0.41	0.52	0.11	0.0330
cg20997710	7				0.47	0.30	0.17	−0.2326
cg03490567	7	*URGCP*	5'UTR;Body	N_Shore	0.23	0.34	0.10	0.0660
cg10218605	7	*PTPRN2*	Body	N_Shore	0.30	0.50	0.20	0.0037
cg00201595	8				0.82	0.78	0.04	−0.3187
cg08092111	8	*RP1*	Body	S_Shore	0.72	0.57	0.16	−0.0906
cg26598831	8				0.63	0.42	0.21	−0.1777
cg06728098	8	*MRPS28*	Body	N_Shore	0.12	0.10	0.02	−0.4359
cg17929627	10	*MKI67*	5'UTR	Island	0.17	0.11	0.06	−2.2937
cg23656300	10				0.71	0.55	0.16	−0.0168
cg20809737	11	*CPT1A*	Body		0.78	0.70	0.09	−0.7679
cg05280814	11			N_Shelf	0.55	0.73	0.18	0.8970
cg15401862	12	*KCNMB4*	3'UTR		0.87	0.80	0.07	−0.2301
cg02767665	12	*TMEM132D*	Body	S_Shore	0.63	0.45	0.18	−0.3594
cg22795345	13	*ARHGEF7*	Body	Island	0.35	0.64	0.29	0.2448
cg14270002	13	*ARHGEF7*	Body	Island	0.28	0.57	0.29	0.2159
cg24743156	14	*CTAGE5*	TSS1500;Body	N_Shore	0.32	0.43	0.11	0.0997
cg25407064	15	*EIF2AK4*	TSS200	Island	0.04	0.03	0.01	−1.5429
cg12921171	15				0.53	0.65	0.12	0.5505
cg18054026	15	*C15orf26*	Body	S_Shore	0.42	0.53	0.12	0.1548
cg11470399	16	*PLK1*	1stExon	Island	0.14	0.11	0.03	−1.5771
cg04138181	16	*PLK1*	Body	S_Shore	0.25	0.15	0.10	−1.1582
cg27106909	16	*YPEL3*	1stExon;5'UTR	N_Shore	0.20	0.37	0.17	0.2890
cg06285575	16	*ZNF267*	Body	S_Shelf	0.21	0.33	0.12	0.0053
cg09848947	16	*FAM38A*	Body		0.77	0.66	0.11	−0.4029
cg06751612	16	*FAM38A*	Body		0.86	0.76	0.11	−0.5474
cg10576459	17				0.89	0.79	0.10	−0.0067
cg01135464	17				0.33	0.57	0.24	0.7303
cg22438006	18			N_Shelf	0.66	0.49	0.18	−0.0625
cg07164161	18	*KCNG2*	Body	S_Shore	0.53	0.42	0.11	−0.0296
cg09960641	19	*LOC100128675*	TSS1500		0.31	0.46	0.14	0.2864
cg25286393	19	*NAPSA*	TSS1500		0.70	0.59	0.11	−0.0201
cg12551567	20	*CDC25B*	1stExon	Island	0.10	0.08	0.02	−0.4047
cg07944494	21			Island	0.94	0.96	0.02	0.1070
cg07260325	X				0.74	0.66	0.08	−0.1695

**Table 2:** Selected characteristics of patients in the Fred Hutchinson prostate cancer patient cohort

Variables	Patients (*n* = 523)		
	Number	Percentage (%)	Mean (SD)
Age at diagnosis (years)			58.1 (7.1)
Race			
African-American	44	8.4	
European-American	479	91.6	
Body mass index at diagnosis (kg/m^2^)			26.9 (3.7)
Pathological stage^a^			
Local	360	68.8	
Regional	163	31.2	
Gleason score			
≤6	252	48.2	
7(3 + 4)	188	35.9	
7(4 + 3)	45	8.6	
8–10	38	7.3	
PSA at diagnosis (ng/mL)^b^			
0−3.9	80	16.2	
4−9.9	313	63.5	
10−19.9	67	13.6	
≥20	33	6.7	
Recurrence^b^			
No recurrence	323	74.9	
Recurrence	108	25.1	
Follow-up time (years)^b^			8.0 (4.2)

*PSA* prostate-specific antigen

^a^Local stage is pT2, N0/NX, M0. Regional stage is pT3-T4 and/or N1, M0

^b^Twenty-six patients had missing data on diagnostic PSA levels, and 92 patients had missing data on recurrence. Three additional patients had missing follow-up data

**Fig. 2:**
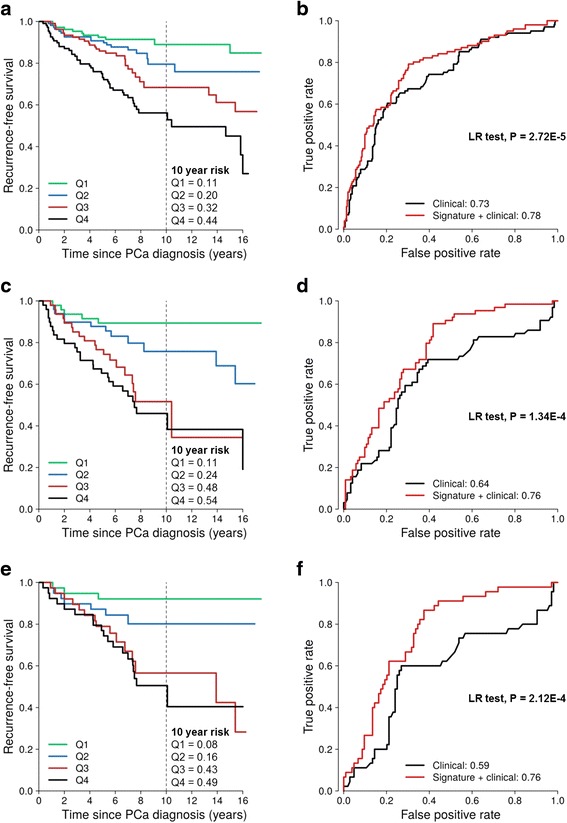
Epigenetic signature of Gleason score and prostate cancer recurrence in the Fred Hutchinson cohort. **a** Recurrence-free survival by quartiles (*Q1–4*) of the epigenetic signature. The *vertical dashed line* shows the recurrence-free survival rate at 10 years after diagnosis. **b** The signature improved the prediction of recurrence beyond the standard clinical-pathological parameters: Gleason score, pathological stage, and diagnostic PSA level (likelihood-ratio (LR) test, *P* < 0.05). **c**, **d** Same analyses as in Fig. 2a, b, but for all patients with Gleason score 7 tumors. **e**–**f** Same analyses as in Fig. 2a, b, but for patients with Gleason score 7(3 + 4) tumors

The epigenetic signature was then tested for its ability to predict recurrence in the FH cohort. Higher levels of the signature were associated with poorer recurrence-free survival (Fig. 2a). The 5- and 10-year risk of recurrence increased with increasing quartiles of the signature: 8, 9, 14, and 27 % and 11, 20, 32, and 44 %, respectively. The HR of recurrence for each 25 % increment in the signature was 1.78 (95 % CI 1.48, 2.16), which remained significant after adjusting for Gleason score, pathological stage, and diagnostic PSA level (Table 3). Adding the signature to a model that included these prognostic parameters significantly improved the AUC for recurrence (0.73 vs. 0.78, *P* = 2.72E−5; Fig 2b). In this study, 24 patients with no recurrence and 17 patients with recurrence had adjuvant therapy after radical prostatectomy. Excluding these 41 patients did not substantially change the signature’s association with recurrence (HR per 25 % increase 1.81; 95 % CI 1.49, 2.20).

**Table 3:** Hazard ratios and 95 % confidence intervals for the association of the epigenetic signature with prostate cancer recurrence

Patients	Analysis model	Variables		Recurrence		
				HR	(95 % CI)	*P* value
All						
	Univariate					
		Signature (per 25 % increase)		1.78	(1.48, 2.16)	2.05E−09
	Multivariate					
		Signature (per 25 % increase)		1.48	(1.21, 1.81)	1.38E−04
		Age (per 1-year increase)		0.98	(0.95, 1.01)	0.21
		Gleason score				
			≤6	1.00		
			7(3 + 4)	2.25	(1.32, 3.48)	2.89E−03
			7(4 + 3)	5.04	(2.64, 9.65)	1.02E−06
			8–10	4.06	(2.00, 8.26)	1.08E−04
		Pathological stage^a^				
			Local	1.00		
			Regional	2.04	(1.33, 3.14)	1.13E−03
		Diagnostic PSA level (per 1-unit increase)		1.00	(1.00, 1.00)	0.92
Patients with Gleason 7 tumors						
	Univariate					
		Signature (per 25 % increase)		1.81	(1.42, 2.31)	1.38E−06
	Multivariate					
		Signature (per 25 % increase)		1.59	(1.24, 2.05)	3.21E−04
		Age (per 1-year increase)		0.99	(0.95, 1.03)	0.56
		Gleason score				
			7(3 + 4)	1.00		
			7(4 + 3)	2.08	(1.18, 3.65)	1.09E−02
		Pathological stage^a^				
			Local	1.00		
			Regional	1.82	(1.06, 3.11)	2.94E−02
		Diagnostic PSA level (per 1-unit increase)		1.00	(0.99, 1.01)	0.82
Patients with Gleason 7(3 + 4) tumors						
	Univariate					
		Signature (per 25 % increase)		1.83	(1.36, 2.45)	5.64E−05
	Multivariate					
		Signature (per 25 % increase)		1.65	(1.21, 2.25)	1.54E−03
		Age (per 1-year increase)		1.00	(0.95, 1.05)	0.86
		Pathological stage^a^				
			Local	1.00		
			Regional	1.83	(0.97, 3.45)	0.06
		Diagnostic PSA level (per 1-unit increase)		1.00	(0.99, 1.01)	0.80

*CI* confidence interval, *HR* hazard ratio, *PSA* prostate-specific antigen

^a^Local stage is pT2, N0/NX, M0. Regional stage is pT3-T4 and/or N1, M0

Patients diagnosed with Gleason score 7 tumors represent a large and clinically heterogeneous subgroup of patients with a variable prognosis [19, 29]. In this study, Gleason 7 patients with the highest levels of the signature had lower recurrence-free survival rates (Fig. 2c; Table 3). Adding the signature to a model with traditional clinical-pathological parameters improved the AUC for recurrence (0.64 vs. 0.76, *P* = 1.34E−4; Fig. 2d). The majority of patients with Gleason score 7 tumors had Gleason 3 + 4, which is considered less aggressive compared to Gleason 4 + 3 [19]. In the subset of patients with Gleason 7(3 + 4) tumors, the signature was associated with a higher risk of recurrence (Fig. 2e; Table 3) and significantly improved the AUC for recurrence (0.59 vs. 0.76, *P* = 2.12E−4; Fig 2f). Although there were few patients with Gleason 7(4 + 3) tumors in the testing dataset, the signature also improved the AUC for recurrence in this subgroup (0.62 vs. 0.70; *P* = 0.14).

For 14 of the genes that encompass CpGs in the signature, DNA methylation levels were associated with mRNA expression levels of the corresponding genes in the FH cohort (*P* < 0.05; Additional file 2: Table S1). For three genes, there was an (modest) inverse correlation between methylation levels of CpGs in gene promoter regions and mRNA expression levels. Three other genes had stronger correlations of higher methylation and lower expression levels (correlation  < −0.2): *ANO7*, *ARHGEF7*, and *PTPRN2*. The CpG sites in these genes were in the gene body or 3′UTR region. Analysis of TCGA data provided confirmatory evidence for these findings (Additional file 2: Table S1).

Correlations between the signature and genome-wide gene expression levels were then evaluated in the FH cohort. We identified 1266 significantly upregulated transcripts (FDR *Q* value < 0.05), which involved 1108 unique genes, and 1673 downregulated transcripts, which involved 1357 unique genes (Additional file 3: Table S2). The genes were used as input for GSEA (Table 4). The analysis showed that the upregulated genes in our study (positive NES) were enriched for gene sets related to cell cycle proliferation (i.e., HALLMARK_E2F_TARGETS, HALLMARK_G2M_CHECKPOINT, HALLMARK_MITOTIC_SPINDLE, and HALLMARK_MYC_TARGETS_V1). Comparing our list of genes to KEGG pathways identified the gene set KEGG_CELL_CYCLE as the top-ranked gene set (NES = 2.36). Finally, we evaluated a 31-gene expression signature of cell cycle proliferation (CCP) that was previously shown to be associated with PCa mortality when assessed in primary tumors [30, 31]. In our study, expression levels of 25 genes in the CCP score significantly increased with higher levels of the epigenetic signature (FDR *Q* value < 0.05).

**Table 4:** Gene Set Enrichment Analysis results

Gene set name	Description	NES
Positive enrichment score		
HALLMARK_E2F_TARGETS	Genes encoding cell cycle-related targets of E2F transcription factors	3.86
HALLMARK_G2M_CHECKPOINT	Genes involved in the G2/M checkpoint, as in progression through the cell division cycle	3.08
HALLMARK_MITOTIC_SPINDLE	Genes important for mitotic spindle assembly	2.19
HALLMARK_EPITHELIAL_MESENCHYMAL_TRANSITION	Genes defining epithelial-mesenchymal transition, as in wound healing, fibrosis, and metastasis	2.15
HALLMARK_ALLOGRAFT_REJECTION	Genes upregulated during transplant rejection	1.96
HALLMARK_MYC_TARGETS_V1	A subgroup of genes regulated by MYC—version 1 (v1)	1.87
Negative enrichment score		
HALLMARK_ANDROGEN_RESPONSE	Genes defining response to androgens	−2.83
HALLMARK_FATTY_ACID_METABOLISM	Genes encoding proteins involved in metabolism of fatty acids	−2.04
HALLMARK_XENOBIOTIC_METABOLISM	Genes encoding proteins involved in processing of drugs and other xenobiotics	−1.98

We identified genes that showed increased expression with higher levels of the epigenetic signature (FDR *Q* < 0.05; *n* = 1108) or decreased expression with higher levels of the signature (FDR *Q* < 0.05; *n* = 1357). These genes were pre-ranked according to Pearson correlation, and this pre-ranked list was used as input for GSEA. The gene sets in the table have an FDR *Q* value < 0.05. A positive value for the NES indicates that higher levels of the signature were associated with increased expression of the genes in a gene set

*FDR* false discovery rate, *NES* normalized enrichment score

## Discussion

In the present study, an epigenetic signature of Gleason score was generated. The study showed that the signature predicted recurrence-free survival after radical prostatectomy.

Gleason score, or grade of the tumor, is the best predictor of PCa prognosis in patients with localized disease [19]. While patients diagnosed with Gleason ≤6 tumors typically have a favorable prognosis, patients with Gleason 8–10 tumors are most likely to experience disease recurrence and progression [19, 32]. We therefore generated a signature by contrasting patients with high (8–10) versus low (≤6) Gleason score tumors. Importantly, the study showed that the signature significantly improved the prediction of recurrence in patients diagnosed with Gleason score 7 tumors. Compared to standard clinical-pathological parameters, the signature improved the AUC for recurrence by 12 %; and for patients with 3 + 4 tumors, there was a 19 % increase in AUC. Patients with Gleason score 7 tumors are clinically heterogeneous [29, 33]. These patients have a variable prognosis, and predicting PCa outcomes is often challenging. The methylation signature may therefore have potential to further improve the prognostication of these patients and might have clinical utility to help guide clinical decision-making (e.g., adjuvant therapy) after radical prostatectomy [34]. Further validation of the signature is however required.

The epigenetic signature, which was created using an agnostic method, includes 52 differentially methylated CpG sites. The genes that encompass CpGs in the signature have roles in different biological pathways including ion channel transport, Akt signaling, and cell cycle, all of which are important for PCa growth. Four genes in the signature encode cell cycle-related targets of E2F transcription factors (*PLK1*, *CDC25B*, *MKI67*, and *RRM2*) [27]. The E2F pathway has a crucial role in cell cycle proliferation and the progression of PCa [35]. We also showed that the methylation levels of several CpGs were associated with mRNA expression levels of the corresponding genes. While the strength of the correlation was modest for most genes, a few genes revealed stronger correlations: *ANO7*, *ARHGEF7*, and *PTPRN2*. The CpGs in these genes were in the gene body or 3′UTR, and higher methylation levels in higher Gleason score tumors were associated with lower mRNA expression of all three genes. Although the link between methylation in these regions of the gene and mRNA expression is not well understood, there is evidence from previous studies that intragenic (gene body) DNA methylation could reduce the efficiency of transcription elongation [36]. The role of *ARHGEF7* and *PTPRN2* in PCa is unknown, but *ANO7* encodes a polytopic membrane protein that is prostate-specific [37], and the methylated CpG site in this gene might therefore be a promising biomarker for more aggressive PCa. The gene is also being studied as a potential target for PCa immunotherapy [38]. Further, a previous study showed that the expression of *ANO7* and *PTPRN2* is downregulated in metastatic prostate tumors [39].

Gene Set Enrichment Analysis showed that the epigenetic signature was associated with increased expression of genes related to cell cycle proliferation. Increased cell proliferation is a key feature of cancer that is required for further neoplastic progression [40]. Previously, Cuzick et al. identified a 31-gene expression score of cell cycle proliferation (CCP) for predicting PCa outcomes [30]. The score is associated with PCa-specific mortality when assessed in primary tumors [30, 31]. In our study, the majority of the genes in the CCP score (*n* = 25) were upregulated with higher levels of the epigenetic signature. In addition, GSEA showed that the signature was associated with decreased expression of androgen-responsive genes. Androgens regulate vital aspects of prostate growth and function [41], and androgen receptor activity inversely correlates with cell cycle proliferation in advanced PCa [42]. Therefore, the epigenetic signature seems to capture important biological pathways and events related to prostate tumor progression.

A number of previous studies on DNA methylation biomarkers for predicting PCa outcomes in patients with localized disease have been conducted. Most of these previous investigations focused on specific candidate genes. Evidence from these studies suggests that hypermethylation of *PITX2*, *GSTP1*, and *APC* is associated with more aggressive PCa and disease prognosis [11, 12]. Some other prior studies have focused on larger sets of CpG sites across the genome and identified different panels of CpG biomarkers for distinguishing more from less aggressive prostate tumors [13–18]. For example, in a previous epigenome-wide analysis from our group, we identified a panel of methylation biomarkers for predicting metastatic-lethal PCa [18]. None of the differentially methylated CpG sites in the prior studies, however, are included in the epigenetic signature of Gleason score. It is important to note that several of the previous studies were limited by small sample size and a limited number of CpG markers evaluated. Additional large investigations of DNA methylation biomarkers for PCa recurrence are therefore needed, including studies to further validate our epigenetic signature and other previously identified prognostic CpG biomarkers.

The present study has important strengths including the relatively large number of patients and the agnostic, genome-wide approach used for building the prognostic epigenetic classifier in the TCGA discovery dataset. The testing cohort has a prospective design with long-term follow-up for patient outcomes. Other prognostic tools (e.g., CAPRA-S, Decipher) were not tested in this study, and additional comparative studies are therefore needed.

## Conclusions

Better tools to identify at the time of diagnosis the subset of PCa patients at the highest risk of recurrence are urgently needed. Our study provides new evidence that DNA methylation profiling has the clinical potential to improve risk prediction for PCa outcomes in patients with clinically localized disease, particularly in patients with Gleason score 7 tumors, which have a variable clinical course and represent a substantial proportion of prostate cancer patients diagnosed worldwide each year.

## Additional files

Additional file 1: Figure S1. Heatmap of the CpG sites included in the epigenetic signature in the Fred Hutchinson cohort. (TIF 16594 kb) Additional file 2: Table S1. Correlations between methylation levels of CpG sites included in the epigenetic signature and mRNA expression levels of corresponding genes. (XLSX 17 kb) Additional file 3: Table S2. Correlations of gene expression levels and the epigenetic signature. (TXT 64 kb)

## Abbreviations

AUC: Area under the curve
CI: Confidence interval
FDR: False discovery rate
FH: Fred Hutchinson
GSEA: Gene Set Enrichment Analysis
HR: Hazard ratio
NES: Normalized enrichment score
PCa: Prostate cancer
PSA: Prostate-specific antigen
ROC: Receiver operating characteristic
TCGA: The Cancer Genome Atlas
