# Supplementary figures and images for: Epigenetic signature of Gleason score and prostate cancer recurrence after radical prostatectomy
Source: Clin Epigenetics. 2016 Sep 15;8:97. doi: 10.1186/s13148-016-0260-z (PMC5024414; doi:10.1186/s13148-016-0260-z)

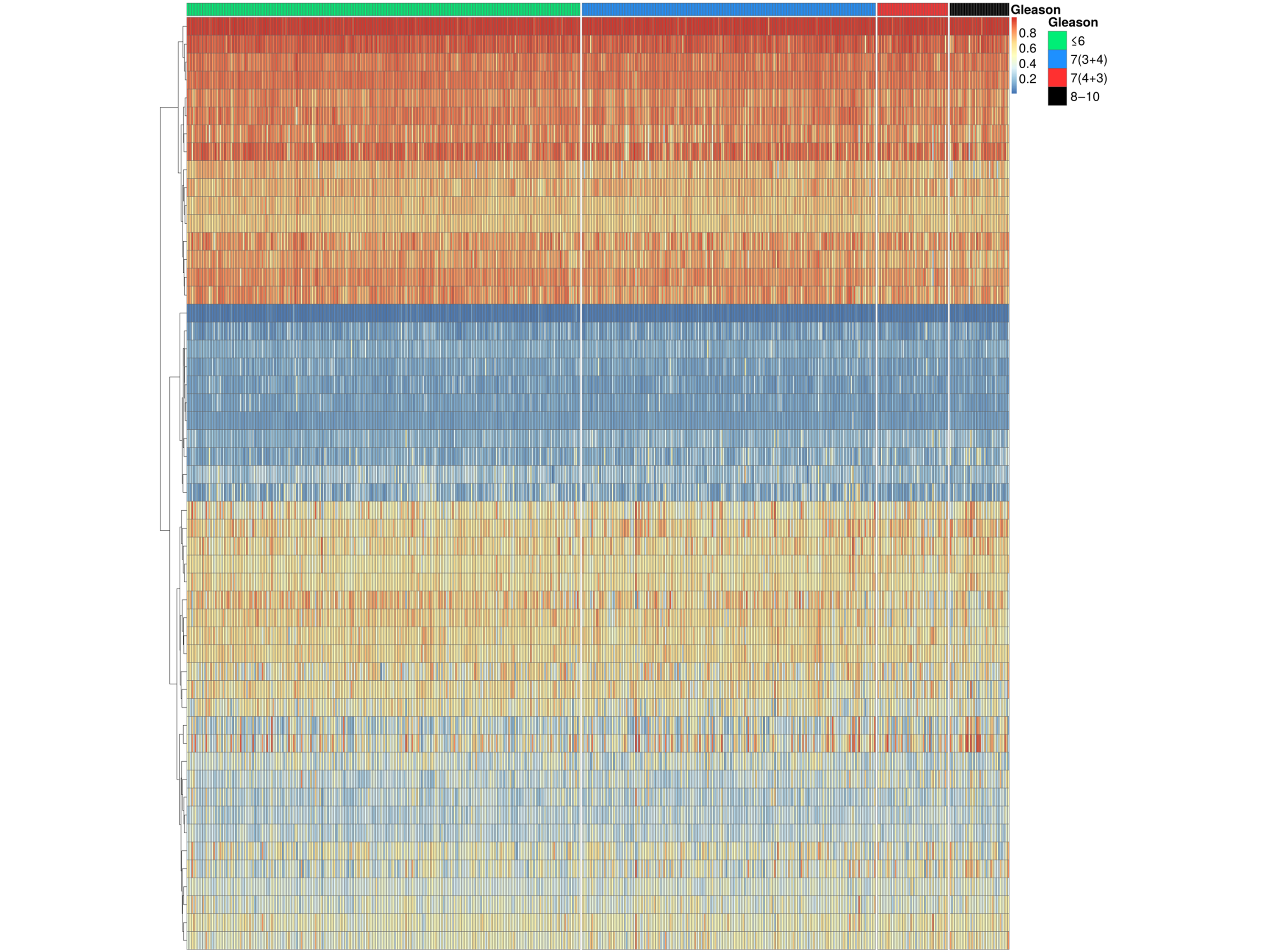

Supplement: Additional file 1: Figure S1. — Heatmap of the CpG sites included in the epigenetic signature in the Fred Hutchinson cohort. (TIF 16594 kb) [file 13148_2016_260_MOESM1_ESM.tif]
